# MicroRNA miR-23b-3p promotes osteosarcoma by targeting ventricular zone expressed PH domain-containing 1 (VEPH1)/phosphatidylinositol 3-kinase/protein kinase B (PI3K/AKT) pathway

**DOI:** 10.1080/21655979.2021.2010383

**Published:** 2021-12-14

**Authors:** Liang Fan, Xing Cao, Yanrong Lei

**Affiliations:** Department of Oncology, The Central Hospital of Wuhan, Tongji Medical College, Huazhong University of Science and Technology, Wuhan, China

**Keywords:** miR-23b-3p, osteosarcoma, veph1, PI3K/AKT, survival, migration

## Abstract

Increasing evidence suggests that dysregulated miRNA expression can lead to the tumorigenesis of osteosarcoma (OS). Nevertheless, the potential role of miR-23b-3p in OS is unclear and remains to be explored. Microarray analysis was performed to identify key genes involved in OS. Reverse transcription quantitative polymerase chain reaction and Western blotting were used to examine miR-23b-3p expression, ventricular zone expressed PH domain-containing 1 (VEPH1) transcript (as well as other transcripts as indicated), and phosphatidylinositol 3-kinase/protein kinase B (PI3K/AKT) signaling pathway-related protein expression. A luciferase reporter gene assay was performed to confirm the regulatory relationship between VEPH1 mRNA and miR-23b-3p. Cell viability was evaluated using the Cell Counting Kit-8 assay, cell growth was assessed using the bromodeoxyuridine enzyme-linked immunosorbent assay, and cell migration was tested using a wound healing assay. We found significant upregulation of miR-23b-3p in OS, which prominently promoted the viability, proliferation, and migration of OS cells. Additionally, VEPH1 was found to be a target of miR-23b-3p and its expression was decreased in OS. Lastly, VEPH1 alleviated the promotion effect of miR-23b-3p on the malignancy phenotypes of OS cells via the PI3K/AKT signaling pathway. Thus, miR-23b-3p augmented the viability, proliferation, and migration of OS cells by directly targeting and downregulating VEPH1, which inhibited the activation of the PI3K/AKT signaling pathway.

## Introduction

As a malignant bone tumor, osteosarcoma (OS) originates directly from osteoid tissues [1]. OS tends to occur in adolescents, and seriously affects their quality of life [2]. Despite recent advances in the treatment of OS, such as radiation and chemotherapy, the prognosis is poor in patients with non-resectable, primary metastatic, or recurrent OS [3]. If not treated in time, there is a high risk of lung metastasis and death [4,5]. Although various studies have investigated the pathogenesis of OS, the molecular mechanism of OS is still unclear. Therefore, it is crucial to identify the potential mechanism of OS and to identify new methods and targets for its treatment.

In recent years, mRNA microarray has been used to screen key genes involved in cancers [6–8]. For instance, GSE87437 downloaded from Gene Expression Omnibus (GEO) datasets was used to analyze differentially expressed genes (DEGs) in OS, and six genes were confirmed as key genes related to OS chemoresistance [9]. Hence, we also used two mRNA microarrays from the GEO datasets to identify DEGs, and ventricular zone expressed PH domain-containing 1 (VEPH1) in two microarrays with low expression attracted our attention. Previous studies have reported that VEPH1 is an intracellular adaptor protein that regulates signaling pathways such as mTOR, TGF-β, AKT, Wnt, FoxO, and Hippo, thus affecting the progression of pathological processes, including cancers [10–12]. Numerous studies have demonstrated that VEPH1 expression is altered in cancers such as hepatocellular carcinoma [11], ovarian cancer [13], colon cancer [14], lung cancer [15], and prostate cancer [16]. These studies showed that VEPH1 might influence the growth and progression of human cancers [17]. Although considerable evidence exits for the differential expression of VEPH1 in different types of cancers, the function and mechanism of VEPH1 in OS are rarely studied and discussed. Therefore, together with our microarray analysis and previous studies, VEPH1 was identified as the gene of interest to be investigated in OS.

Mature microRNAs (miRNAs) are approximately 19‒25 nucleotides in length, encoded by endogenous genes and belong to a non-coding RNA family [18]. miRNAs are the most studied non-coding RNAs, especially in human cancers [19]. In general, miRNAs exert their regulatory roles in the transcription and/or translation of target genes by specifically combining with the 3ʹ untranslated regions (3ʹ-UTRs) of the downstream target genes, thereby reducing their expression [20,21]. miRNAs exert their carcinogenic or tumor-suppressive effects by binding and inhibiting downstream target genes [22]. Therefore, miRNAs can be used as effective biomarkers and drug targets for the diagnosis and treatment of cancer, such as pancreatic cancer [23]. Hence, we used starBase and TargetScan to predict the miRNAs that could bind to the VEPH1 gene. Finally, miR-23b-3p was identified as a miRNA of interest based on the screening results and its abnormal expression in different cancers [24–28].

Phosphatidylinositol 3-kinase (PI3K) has been found to phosphorylate inosine phosphate, which is involved in the production of D3-phosphoinosine by regulating proteins such as protein kinase B (PKB or AKT), and promotes a variety of cellular functions, including differentiation, maturation, motility, and survival [29]. Previous studies have reported that PI3K/AKT signaling is frequently upregulated in various cancer types and plays a key role in the growth and survival of OS cells [30,31]. Therefore, it is necessary to explore the regulatory mechanism of the PI3K/AKT pathway in OS.

This study examined the functions of VEPH1, miR-23b-3p, and PI3K/AKT signaling pathways in OS and explored the relationship between VEPH1, the PI3K/AKT signaling pathway, and miR-23b-3p. The present study enriched the knowledge on miR-23b-3p/VEPH1/PI3K/AKT as well as their regulatory relationship in OS. Our findings may help uncover miR-23b-3p/VEPH1/PI3K/AKT as new drug targets for the treatment of OS.

## Materials and methods

### Microarray analysis

Two mRNA microarray profiles were downloaded from the GEO datasets (https://www.ncbi.nlm.nih.gov/gds/?term=). GSE11414 included two normal human osteoblast and four OS cell line samples, and GSE30807 included one sample each of normal mesenchymal stem cell and OS cell. Differentially expressed genes (DEGs) were identified using GEO2R built from the GEO datasets. Overlapping DEGs with adj.P value less than 0.01 (GSE11414) or less than 0.05 (GSE30807) and log fold change (logFC) less than −1.5 were selected. TargetScan Human 7.2 (http://www.targetscan.org/vert_72/) and starBase database (http://starbase.sysu.edu.cn/) were used to select the miRNAs that could bind to the gene of interest [32].

### Clinical tissue samples

OS and adjacent non-tumor bone tissues were acquired from 24 patients diagnosed with OS at our hospital. None of the patients received any anticancer treatment prior to the surgical resection. OS was classified according to the World Health Organization (WHO) classification system [33]. All patients agreed for the acquisition of clinical samples and their participation in the study and provided their informed consent. This study was approved by the ethics committee of our hospital (approval number: 2019–5). After surgical excision, the tissue samples were immediately stored in liquid nitrogen for subsequent studies. The clinical characteristics of the 24 patients with OS are shown in Table 1.

**Table 1.** Clinical characteristics of 24 cases osteosarcoma patients

characteristics	N = 24	miR-23b-3p expression		P value
		Low (N = 12)	High (N = 12)	
Age(years)				0.400
≥11	9	6	3	
<11	15	6	9	
Gender				0.414
Male	11	4	7	
Female	13	8	5	
Location				0.241
Distal femur	11	7	4	
Proximal tibia	6	2	4	
Proximal humerus	4	3	1	
Proximal femur	2	0	2	
Other	1	0	1	
Recurrence				0.037
Yes	5	0	5	
No	19	12	7	
Metastasis				0.012
Yes	13	3	10	
No	11	9	2	
Death				0.036
Yes	10	2	8	
No	14	10	4	
WHO classification				0.080
Osteoblastic	11	8	3	
Chondroblastic	9	2	7	
Fibroblastic	4	2	2	

### Cell lines and cell culture

All cell lines, except for the HOS cell line, including the human osteoblast hFOB1.19 and three OS cell lines (SJSA-1, Saos-2, and U2OS) were purchased from the BeNa Culture Collection (BNCC, China). The HOS cell line was purchased from the American Type Culture Collection (ATCC, Cat#: CRL-1543, USA). All cells were cultured in Dulbecco’s modified Eagle’s medium (DMEM) (Gibco, USA) supplemented with 10% fetal bovine serum (FBS) (Gibco), 100 U/mL streptomycin, and penicillin (Invitrogen, USA). All cell lines were placed in a 5% CO_2_ incubator at 37°C.

### Construction of VEPH1 overexpression vector

VEPH1 overexpression vectors (VEPH1-OE) were purchased from GenePharma (Shanghai, China). Briefly, the total RNA was extracted from HOS cells and reverse transcribed into cDNA as a template to construct VEPH1-OE vectors. Then, full-length VEPH1 containing the binding site of miR-23b-3p was amplified by polymerase chain reaction (PCR). Then, the PCR products were cloned between the EcoRV and Xhol sites of the pcDNA 3.1(+) vector (Geenseed Biotech, China) to construct VEPH1 overexpression vectors, which were used to transfect cells. The stably transfected clones were selected using G418 (Promega, USA) after transfection. The pcDNA 3.1(+) vector-transfected cells were used as controls.

### Cell transfection and treatment

MiR-23b-3p inhibitor, miR-23b-3p mimic, and miRNA negative control (NC) of miR-23b-3p were purchased from GenePharma. The HOS and U2OS cells were transfected with 40 nM miR-23b-3p inhibitor, miR-23b-3p mimic, NC, or VEPH1-OE vectors using Lipofectamine 3000 and RNAiMax (Life Technologies, USA) according to the manufacturer’s instructions. Transfection efficiency was confirmed by reverse transcription quantitative (RT-q) PCR 48 h after cell transfection. The primer sequences used are listed in Table S1. To block the PI3K/AKT signaling pathway, HOS and U2OS cells were treated with 10 μM LY294002 (the PI3K/AKT pathway inhibitor; Sigma-Aldrich, USA) for 48 h [34].

### RT-qPCR

Total RNA from frozen tissues or cultured cells was isolated and purified using TRIzol reagent (Invitrogen) following the manufacturer’s instructions. The RNA concentration and purity were measured using a spectrophotometer (METASH, China). Then, 1 μg of total RNA was reverse transcribed into cDNA using SuperScript IV VILO Master Mix (Invitrogen) following the manufacturer’s instructions. RT-qPCR was performed using PowerUp SYBR Green Master Mix (Invitrogen). Uracil6 (U6) acted as the internal reference for miR-23b-3p, whereas glyceraldehyde-3-phosphate dehydrogenase (GAPDH) served as an internal reference for the mRNAs. Relative RNA expression was determined using the 2^−ΔΔCt^ method [35]. All reactions in this experiment were performed in triplicate; the primer sequences are listed in Table 2.

**Table 2.** The primer sequences for RT-qPCR

Name	Primer sequences (5ʹ-3ʹ)
miR-23b-3p	Forward: ACACTCCAGCTGGGATCACAT TGCCAGGGAT
	Reverse: CTCAACTGGTGTCGTGGAGTCGGCAATTCAG TTGAGGTGGTAAT
U6	Forward: CTCGCTTCGGCAGCACA
	Reverse: AACGCTTCACGAATTTGCGT
VEPH1	Forward: CAGAATATATCTGATGCCCACAAAA
	Reverse: CAGGATGGAGAGTTCAGGCA
GAPDH	Forward: CAAGGCTGAGAACGGGAAG
	Reverse: TGAAGACGCCAGTGGACTC

### Cell counting kit-8 (CCK-8) assay

CCK-8 (Dojindo, Japan) was used to measure cell viability in HOS and U2OS cells according to the manufacturer’s protocol. Briefly, transfected HOS and U2OS cells (1 × 10^3^/well/100 μL) were inoculated into 96-well plates during the logarithmic growth period and then placed in an incubator (37°C, 5% CO_2_) for 0, 24, 48, and 72 h. After adding 10 µL of CCK-8 solution to each well, the cells were cultured under the same conditions for 2 h. Subsequently, cell viability was evaluated by measuring the absorbance at 450 nm using a microplate reader. Each experiment was performed in triplicate [36].

### Bromodeoxyuridine enzyme-linked immunosorbent assay (BrdU-ELISA)

BrdU ELISA was performed to measure cell proliferation in HOS and U2OS cells using the CytoSelect™ BrdU Cell Proliferation ELISA Kit (Cat#CBA-251, Cell Biolabs, USA). Briefly, transfected HOS and U2OS cells (1 × 10^5^/well/100 μL) were inoculated into 96-well plates and incubated for 48 h in an incubator (37°C, 5% CO_2_). After adding 10 µL of 10× BrdU solution to each well, the cells were cultured under the same conditions for 4 h. After washing with PBS, the cells were incubated in 100 µL Fix/Denature Solution at 37°C for 30 min to fix and denature cellular DNA. Next, the cells were incubated in 100 µL diluted anti-BrdU antibody, followed by incubation in horseradish peroxidase (HRP)-coupled secondary antibody diluent. After reacting with the substrate, cell proliferation was evaluated by measuring the absorbance at 450 nm using a microplate reader. Each experiment was performed in triplicate [37].

### Wound healing assay

A wound healing assay was performed to assess cell migration. HOS and U2OS cells (5 × 10^4^) were inoculated into each well of the 6-well plates at 37°C in an atmosphere of 5% CO_2_ to form a confluent monolayer. A wound gap was scratched in the middle of the cell layer using a sterile 20-µL micropipette. The wound was then washed and the cell debris removed using phosphate-buffered saline (PBS). After replacing the serum-free medium, the cells were cultured for another 1 day. Cells gradually migrated to the wound surface and were photographed at 0 and 24 h using an inverted microscope. The migration rate was calculated to represent the migration ability of cells in each group as the ratio of the difference in wound gap width between 0 h and 24 h to the wound gap width at 0 h [38].

### Dual-luciferase reporter gene assay

We mutated the VEPH1 3ʹ-UTR sequences to which miR-23b-3p could bind. Then, the wild-type or mutant VEPH1 3ʹ-UTR sequences were inserted into the pGL4 luciferase reporter vector (Promega). The HOS and U2OS cells were co-transfected with the recombinant reporter plasmids and the miR-23b-3p mimic. After 48 h of transfection, the cultured cells were collected and subjected to the Dual-Luciferase® Reporter Assay System (Promega, E1910) following the manufacturer’s instructions. Renilla luciferase and firefly luciferase activity were sequentially measured in each sample using a luminometer. Renilla luciferase activity was used as an internal reference to normalize the relative activity of firefly luciferase [39].

### Western blot assay

Western blot analysis was performed as described previously [40]. After transfection, the HOS and U2OS cells were collected and radioimmunoprecipitation assay (RIPA) lysis buffer was added to lyse the cells and isolate the protein. After centrifugation, the supernatant containing the total protein was collected. Then, protein concentration was measured using the bicinchoninic acid (BCA) method. Proteins were separated using 10% sulfate-polyacrylamide gel electrophoresis (SDS-PAGE) gel and transferred to polyvinylidene fluoride (PVDF) membranes (Millipore, USA). The membranes were blocked with skim milk and then incubated with primary antibodies against VEPH1 (Cat#: ab121875, Abcam, UK) and GAPDH (Cat#: ab181602, Abcam). After overnight incubation, the membranes were incubated with secondary antibodies. Finally, an electrogenerated chemiluminescence (ECL) detection kit (Pierce Biotechnology, USA) was used to detect the protein bands. Protein expression was quantified using FluorChem FC2 (Alpha Innotech, USA).

### Statistical analysis

The results were analyzed using the SPSS software (version 20.0; SPSS, USA). The mean ± standard deviation values represent three independent experiments. Student’s *t*-test and analysis of variance (ANOVA) were used to determine significant differences between two or multiple groups. Dunnett’s or Tukey’s multiple comparison tests were performed as part of one- or two-way ANOVA. Statistical significance was set at p < 0.05.

## Results

Here, we aimed to explore valuable molecular therapeutic targets for OS. We hypothesized that miR-23b-3p participated in OS promotion by downregulating VEPH1 and activating the PI3K/Akt signaling pathway. Clinical analysis showed that miR-23b-3p was upregulated in OS and correlated with tumor recurrence and metastasis. In addition, miR-23b-3p overexpression promoted the survival and migration of OS cells, whereas miR-23b-3p interference had the opposite effect. Moreover, the expression of VEPH1 in OS was negatively regulated by miR-23b-3p and blocked the activation of the PI3K/AKT signaling pathway. Therefore, the miR-23b-3p/VEPH1/PI3K/AKT signaling axis has a certain value in OS.

### Increased expression of miR-23b-3p in OS

**Figure 1.**
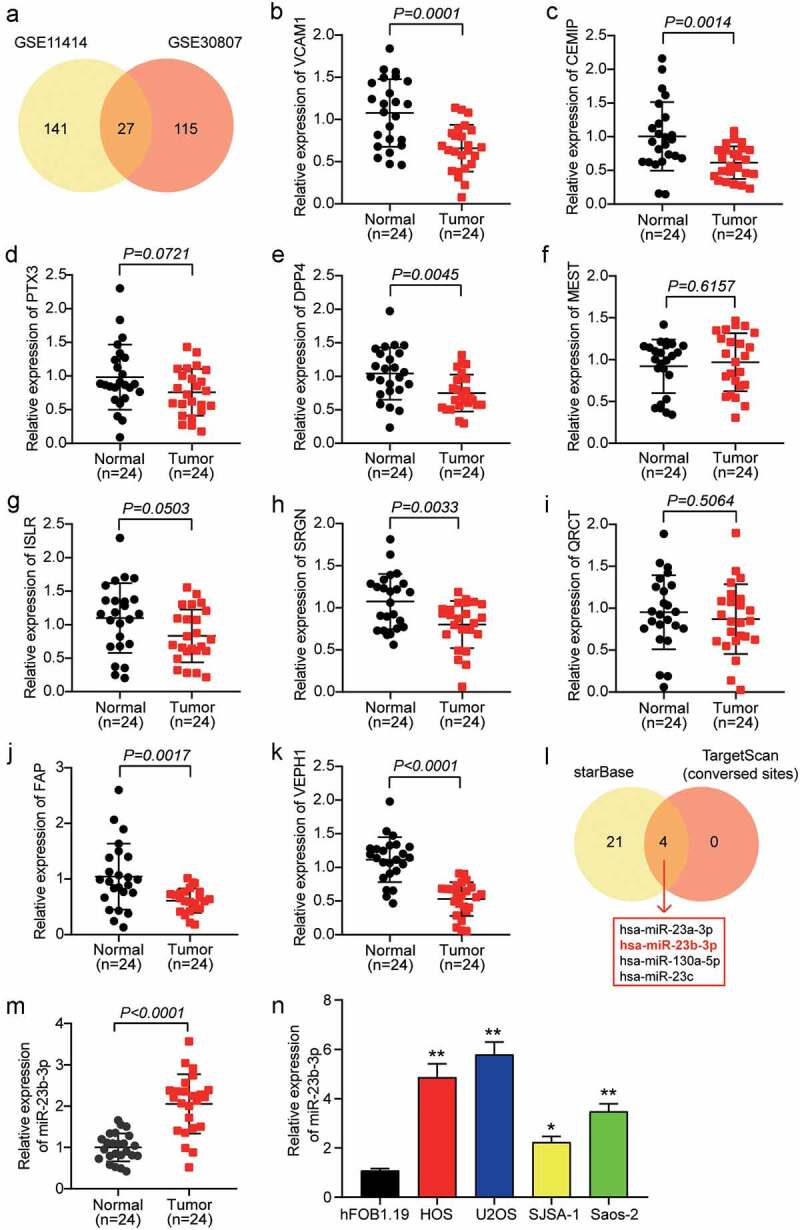
**MiR-23b-3p was up-regulated in OS cells and tissues**. (a) The overlapping downregulating DEGs were selected out from GSE11414 and GSE30807. (b-k) The expression of top 10 downregulating DEGs were measured in tumor tissues and adjacent healthy tissues by RT-qPCR. n = 19, Student’s *t-*test. (l) ENCORI starBase and TargetScan Human 7.2 algorithms were used to predict the miRNAs that could bind to VEPH1. (m) The relative expression of miR-23b-3p was detected by RT-qPCR in tumor tissues and adjacent healthy tissues. n = 24, Student’s *t-*test. (n) The relative expression of miR-23b-3p was detected by RT-qPCR in various human OS cell lines (HOS, U2OS, SJSA-1 and Saos-2) and normal cell line (hFOB1.19). *P < 0.05, **P < 0.001 compared with hFOB1.19 cell line, ANOVA. Each bar represents the mean ± SD of at least three independent experiments for each group

To identify the key genes involved in OS, we used two mRNA microarray datasets, GSE11414 and GSE30807. A total of 168 DEGs were selected from GSE11414 with an adj.P value less than 0.01 and logFC less than −1.5, and 142 DEGs were selected from GSE30807 with a adj.p value less than 0.05 and logFC less than −1.5. Twenty-seven overlapping DEGs were selected from both GSE11414 and GSE30807 (Figure 1a). We identified the top 10 downregulated genes in our clinical tissue samples (n = 24) and found that six genes were significantly downregulated in OS tissues with p values less than 0.05 (Figure 1b–k). Among the six downregulated genes, VEPH1 was the most downregulated gene in OS tissues. To identify the miRNAs that might affect OS development by targeting VEPH1, ENCORI starBase, and TargetScan Human 7.2 algorithms were used to predict the miRNAs that could bind to VEPH1. This analysis showed that four miRNAs, hsa-miR-23a-3p, hsa-miR-23b-3p, hsa-miR-130a-5p, and hsa-miR-23 c, were the overlapping miRNAs (Figure 1l). Previous studies have reported that miR-23a-3p expression decreased and played a negative role in OS [41] and that miR-130a-5p and miR-23 c were proven to be tumor suppressors in multiple cancers [24,42–44]. In contrast to miR-23a-3p, miR-130a-5p, and miR-23 c, miR-23b-3p was shown to be upregulated and promoted the progression of OS [28], which is the opposite of the effect of VEPH1. Therefore, we identified miR-23b-3p as the miRNA of interest. We then examined the expression of miR-23b-3p in 24 pairs of OS and adjacent healthy tissue samples. We found that there was a 2-fold increase in miR-23b-3p levels in OS tissues compared with normal tissues (Figure 1m). Moreover, we found that miR-23b-3p expression was positively correlated with recurrence, metastasis, and death, but not with gender, age, location, or WHO classification (Table 1). Similar to tissues, miR-23b-3p expression in the OS cell lines, including U2OS, SJSA-1, HOS, and Saos-2, especially in the two OS cell lines, HOS and U2OS, was significantly higher than that in the non-cancer cell line hFOB1.19 (Figure 1n). Therefore, the HOS and U2OS cell lines were selected for cell functional experiments.

### MiR-23b-3p promotes cell viability, proliferation, and migration in OS

**Figure 2.**
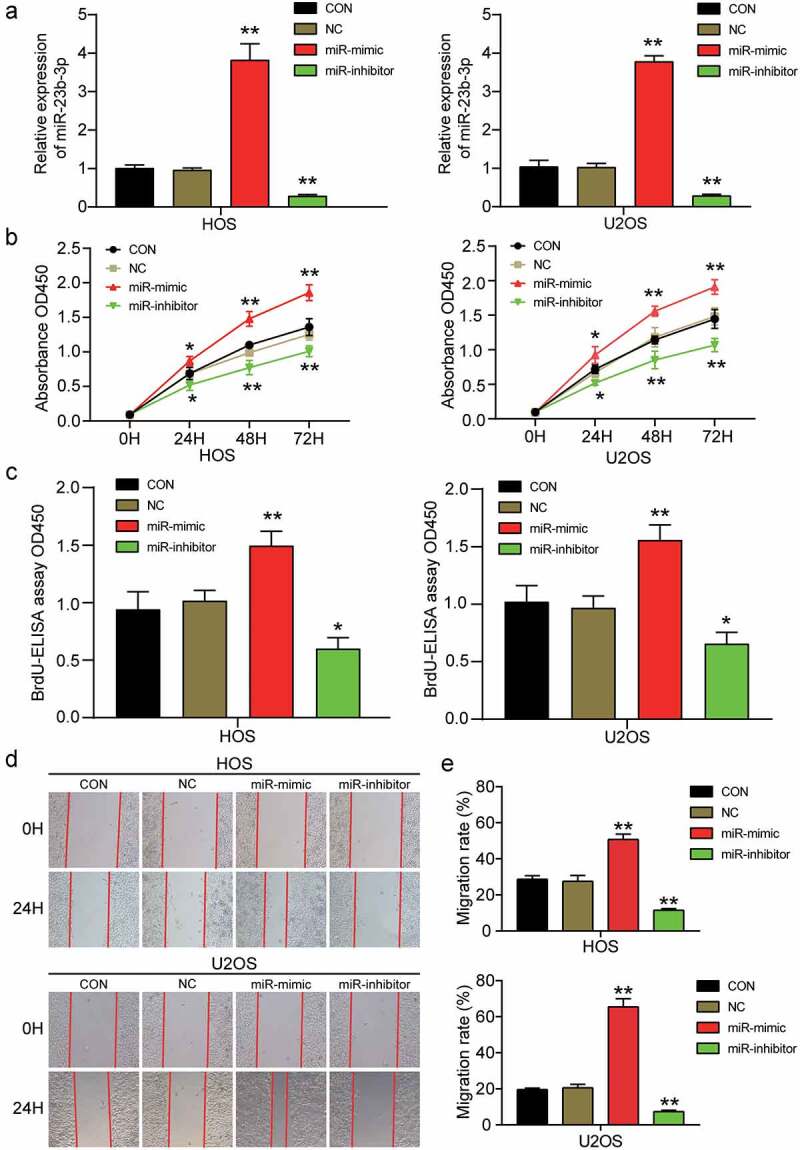
**MiR-23b-3p promoted the cell viability, proliferation and migration in OS**. (a) The transfection efficiency of miR-23b-3p mimic and miR-23b-3p inhibitor were ascertained by RT-qPCR analysis using U6 as the internal control. (b) CCK-8 assay was performed to determine the cell viability in HOS and U2OS cells after treatment with miR-23b-3p mimic, miR-23b-3p inhibitor or negative control. (c) The effect of miR-23b-3p on cell proliferation was evaluated by BrdU-ELISA assay in HOS and U2OS cells after treatment with miR-23b-3p mimic, miR-23b-3p inhibitor or negative control. (d-e) After transfection with miR-23b-3p mimic, miR-23b-3p inhibitor or negative control for 48 h, HOS and U2OS cells were subjected to wound healing assays and images were taken at 0 and 24 h (×100). Representative images were shown in Figure 2d and the migration rate was shown in Figure 2e. Data were from three independent experiments and presented as the mean ± SD. miR-mimic, miR-23b-3p mimic. miR-inhibitor, miR-23b-3p inhibitor. *P < 0.05, **P < 0.001 compared with control group, ANOVA

To further study the effect of miR-23b-3p in OS, we transfected miR-23b-3p mimic and inhibitor into HOS and U2OS cells to upregulate or downregulate the expression of miR-23b-3p. After determining the transfection efficiency of the mimic and inhibitor by RT-qPCR, we found that miR-23b-3p expression in the miR-23b-3p mimic group was approximately three times higher than that in the control group, whereas miR-23b-3p expression in the miR-23b-3p inhibitor group was suppressed by more than 70% compared to that in the control group (Figure 2a). Subsequently, the miR-23b-3p mimic and inhibitor were transfected into HOS and U2OS cells to explore the influence of miR-23b-3p upregulation or downregulation on the viability, proliferation, and migration of OS cells. The CCK-8 assay showed that cell viability was markedly enhanced by the miR-23b-3p mimic and efficiently inhibited by the miR-23b-3p inhibitor in HOS and U2OS cells (Figure 2b). The BrdU ELISA assay showed that the miR-23b-3p mimic led to a 1.5-fold increase in optical absorbance in both HOS and U2OS cell lines, whilst the miR-23b-3p inhibitor led to an almost 50% decrease in absorbance, indicating that miR-23b-3p contributed to cell proliferation (Figure 2c). Subsequently, cell migration was evaluated using a wound healing assay; it was found that the migration rate was increased by approximately 1.9-fold after transfection with miR-23b-3p mimic, whereas the migration rate declined by approximately 55% after HOS and U2OS cells were transfected with the miR-23b-3p inhibitor (Figure 2(d,e)). These results revealed that upregulation of miR-23b-3p could enhance the migration and proliferation of OS cells, whereas a downregulation of miR-23b-3p decreased the migration and proliferation of OS cells.

### VEPH1 is a downstream target of miR-23b-3p

**Figure 3.**
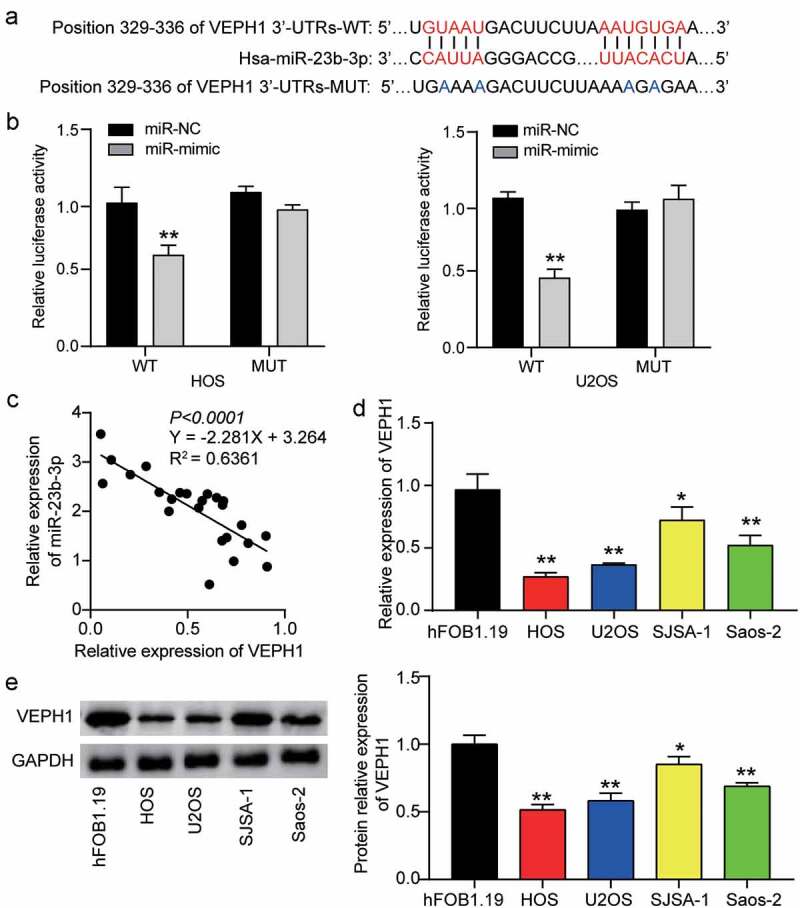
**MiR-23b-3p directly bound to VEPH1**. (a) The predicted miR-23b-3p binding sites in the 3ʹ-UTR of VEPH1 mRNA by TargetScan. 3ʹUTRs: 3ʹUTR sequence. (b) HOS and U2OS cells were co-transfected with miR-23b-3p mimics (or NC) and luciferase reporter plasmid containing wide-type or mutant VEPH1 3ʹ-UTR, and the luciferase activities were detected to confirm the direct target site. **P < 0.01 compared with the co-transfection of miR-NC and wild-type VEPH1, ANOVA. (c) The correlation between the expression of VEPH1 and that of miR-23b-3p in OS tissues. (d) The relative expression of VEPH1 was detected by RT-qPCR in various human OS cell lines (HOS, U2OS, SJSA-1 and Saos-2) and normal cell line (hFOB1.19).*P < 0.05, **P < 0.01 compared with hFOB1.19 cells, ANOVA. (e) The expression of VEPH1 protein was detected by Western blot assay in various human OS cell lines (HOS, U2OS, SJSA-1 and Saos-2) and normal cell line (hFOB1.19). *P < 0.05, **P < 0.01 compared with hFOB1.19 cells, ANOVA. Data were from three independent experiments and presented as the mean ± SD

To further understand the underlying mechanism of miR-23b-3p in OS, we used TargetScan to determine the potential target of miR-23b-3p. This analysis showed that the 3ʹ-UTR of VEPH1 mRNA contained the binding site of miR-23b-3p (Figure 3a). Subsequently, we confirmed this prediction by performing a dual-luciferase reporter gene assay. Analysis of the luciferase activity revealed that upregulation of miR-23b-3p clearly reduced luciferase activity in the wild-type VEPH1 group, but not in the mutant-type VEPH1 group, suggesting a close link between miR-23b-3p and VEPH1 mRNA (Figure 3b). In addition, Pearson correlation analysis revealed a negative correlation between VEPH1 mRNA expression and miR-23b-3p expression in OS tissues (Figure 3c). Similarly, we found lower VEPH1 expression in a range of OS cells (U2OS, SJSA-1, HOS, and Saos-2) than in the non-cancer cell line (hFOB1.19) (Figure 3d). The Western blot assay showed a similar trend: the expression of VEPH1 protein was reduced in OS cells, especially in HOS and U2OS cells (Figure 3e). Together, these data revealed that miR-23b-3p might directly target VEPH1 mRNA and negatively regulate VEPH1 expression in human OS.

### MiR-23b-3p promotes migration of OS cells by inhibiting VEPH1

**Figure 4.**
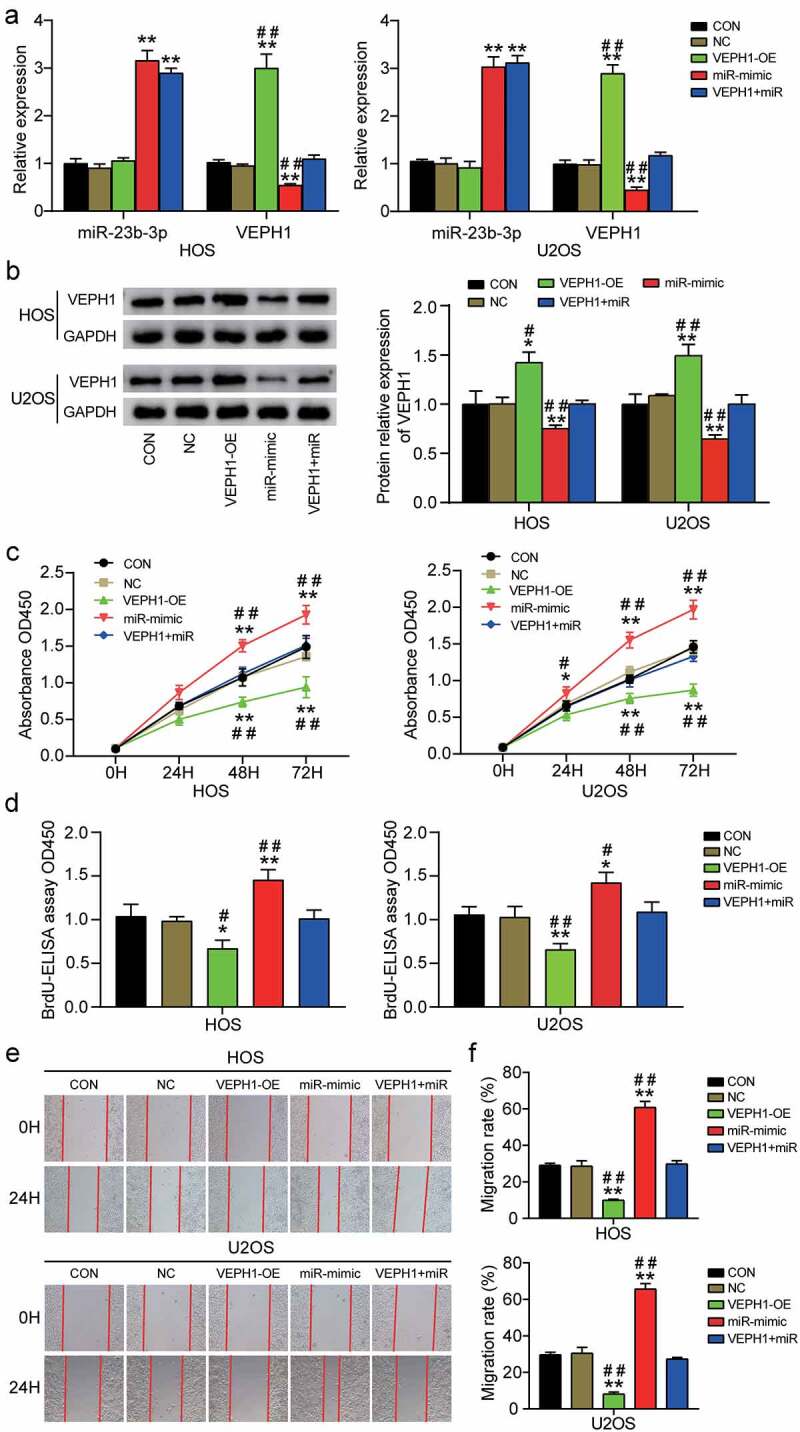
**MiR-23b-3p promoted the cell viability, proliferation and migration by inhibiting the expression of VEPH1 in OS**. (a) The relative expression of miR-23b-3p and VEPH1 mRNA were detected by RT-qPCR in HOS and U2OS cells after transfection with miR-23b-3p mimic, VEPH1 OE, or both. (e) The expression of VEPH1 protein was detected by Western blot assay in HOS and U2OS cells after transfection with miR-23b-3p mimic, VEPH1 OE, or both. (c) CCK-8 assay was performed to determine the cell viability in HOS and U2OS cells after transfection with miR-23b-3p mimic, VEPH1 OE, or both. (d) Cell proliferation abilities were evaluated by BrdU-ELISA assay in HOS and U2OS cells after transfection with miR-23b-3p mimic, VEPH1 OE, or both. (e-f) Cell migration abilities were evaluated by wound healing assay in HOS and U2OS cells after transfection with miR-23b-3p mimic, VEPH1 OE, or both. Representative images were shown in Figure 4e and the migration rate were shown in figure 4f. Data were from three independent experiments and presented as the mean ± SD. VEPH1-OE, VEPH1 overexpression vectors. miR-mimic and miR, miR-23b-3p mimic. *P < 0.05, **P < 0.01 compared with control group, ^#^P < 0.05, ^##^P < 0.01 compared with the co-transfection of miR-23b-3p mimic and VEPH1 OE group, ANOVA

To further investigate how miR-23b-3p promotes the viability, proliferation, and migration of OS cells, we conducted rescue experiments. First, we transfected VEPH1-OE, miR-23b-3p mimic, or both into HOS and U2OS cell lines. As can be seen from the results of RT-qPCR, VEPH1-OE obviously increased the mRNA levels of VEPH1 by 3-fold in HOS or U2OS cells. However, it is worth noting that upregulated miR-23b-3p had a clear inhibitory effect on VEPH1 expression, whereas overexpression of VEPH1 had no apparent effect on miR-23b-3p expression (Figure 4a). Western blot analysis showed that the expression of VEPH1 protein increased by approximately 1.5-fold in the VEPH1-OE group, whereas the miR-23b-3p mimic led to an approximately 60% decrease in the expression of VEPH1 protein (Figure 4b). The expression of VEPH1 in the co-transfection group was not significantly different from that in the control group. Subsequently, we conducted the CCK-8 assay to evaluate whether VEPH1 affects miR-23b-3p-induced cell viability. The results showed that the viability of HOS and U2OS cells was repressed by VEPH1 overexpression and the promoting function of miR-23b-3p in cell viability was reversed by co-transfection with VEPH1-OE and miR-23b-3p mimic (Figure 4c). Similar to cell viability, the proliferation of HOS and U2OS cells was also suppressed by VEPH1 overexpression, whereas co-transfection alleviated miR-23b-3p-induced cell proliferation (Figure 4d). Additionally, the outcomes of the wound healing assay showed that the cell migration rate decreased by almost 50% after VEPH1-OE transfection and that the overexpression of VEPH1 significantly overturned the promoting function of miR-23b-3p in cell migration after co-transfection with VEPH1-OE and miR-23b-3p mimic (Figure 4e–f). Overall, these findings revealed that VEPH1 is a tumor suppressor gene, whose expression is suppressed by miR-23b-3p in OS and that VEPH1 could partially attenuate the promoting function of miR-23b-3p in OS cell viability, proliferation, and migration.

### MiR-23b-3p targeting of VEPH1 promotes OS through the PI3K/AKT pathway

**Figure 5.**
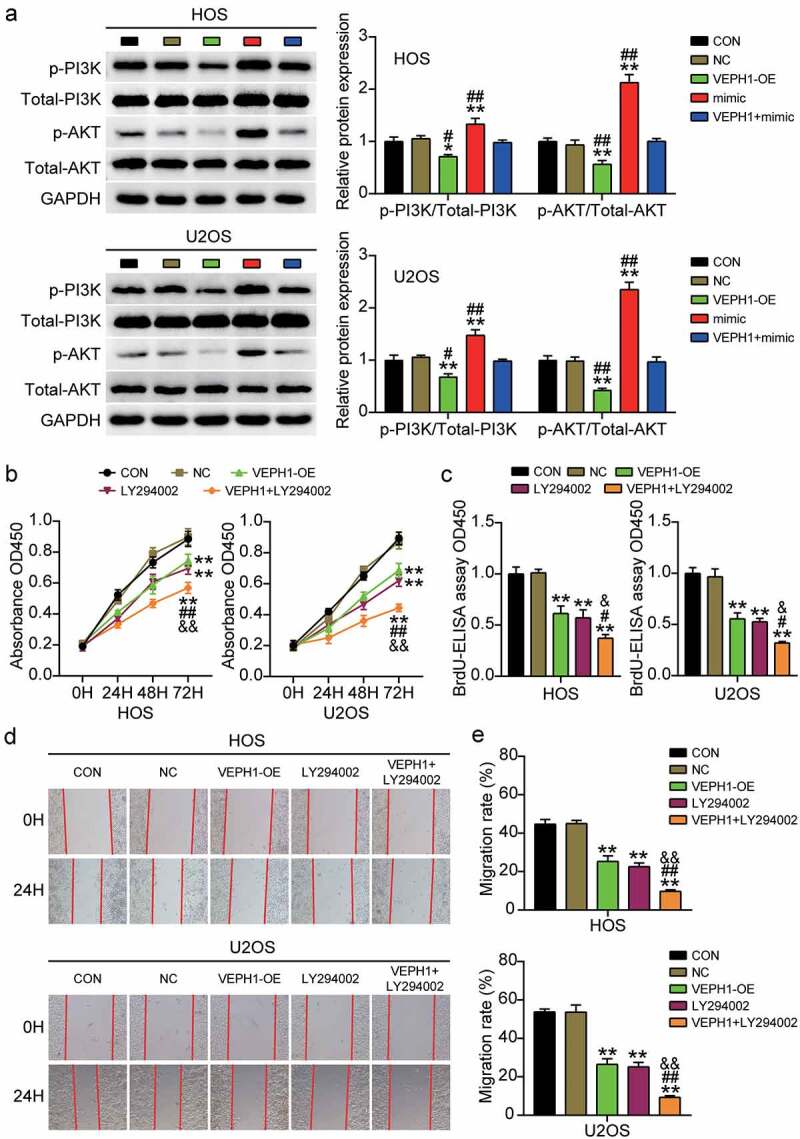
**MiR-23b-3p targeting VEPH1 promotes OS through the PI3K/AKT pathway**. (a) The expression of p-PI3K and p-AKT protein was detected by Western blot assay in HOS and U2OS cells after transfection with miR-23b-3p mimic, VEPH1 OE, or both. (b) CCK-8 assay was performed to determine the cell viability in HOS and U2OS cells after transfection with VEPH1 OE or treated with LY294002. (c) Cell proliferation abilities were evaluated by BrdU-ELISA assay in HOS and U2OS cells after transfection with VEPH1 OE or treated with LY294002. (d-e) Cell migration abilities were evaluated by wound healing assay in HOS and U2OS cells after transfection with VEPH1 OE or treated with LY294002. Representative images were shown in Figure 5d and the migration rate were shown in Figure 5e. Data were from three independent experiments and presented as the mean ± SD. VEPH1-OE, VEPH1 overexpression vectors. LY294002, PI3K/AKT pathway inhibitor. *P < 0.05, **P < 0.01 compared with control group, ^#^P < 0.05, ^##^P < 0.01 compared with the co-treatment of LY294002 and VEPH1 OE group, ANOVA

The PI3K/AKT pathway plays an important role in the malignant behavior of tumor cells; therefore, we investigated the regulatory effect of the PI3K/AKT pathway in OS. After miR-23b-3p or VEPH1 overexpression, we examined the expression levels of important molecules in the signaling pathway in HOS and U2OS cells. The results showed that overexpression of VEPH1 inhibited the phosphorylation of PI3K and AKT and reversed the miR-23b-3p mimic-induced upregulation of p-PI3K and p-AKT (Figure 5a). In addition, OS cells were treated with PI3K/AKT pathway inhibitor (LY294002) or overexpressed for VEPH1 to explore the changes in cell survival and migration. CCK-8 and BrdU assays showed that LY294002 treatment inhibited the viability and proliferation of HOS and U2OS cells and further aggravated the cell survival inhibition caused by the overexpression of VEPH1 (Figure 5(b,c). Moreover, the results of the wound healing assay showed that the cell migration rate decreased after the PI3K/AKT pathway was blocked and the inhibition of cell migration by VEPH1-OE was enhanced (Figure 5(d–e)). These results suggest that miR-23b-3p targets VEPH1 and promotes the viability, proliferation, and migration of OS cells via the PI3K/AKT pathway.

## Discussion

OS, a malignant bone tumor, occurs mostly in adolescents. Currently, the therapeutic outcomes of OS are poor, leading to the impairment of the quality of life and death of many children and adolescents [45]. In the current study, VEPH1 and miR-23b-3p were first confirmed as a key gene and miRNA involved in OS. Then, miR-23b-3p was identified as a tumor promoter and positively regulated not only in OS tissues but also in OS cells. Notably, upregulation of miR-23b-3p significantly promoted the viability, proliferation, and migration of OS cells. Moreover, VEPH1 was considered to be a new direct target of miR-23b-3p in OS, and its expression was markedly reduced in OS, showing a negative correlation with miR-23b-3p. In addition, we showed that miR-23b-3p could contribute to cell viability, cell proliferation, and cell migration in OS by targeting and downregulating VEPH1, which could be both a new biomarker and drug target in the treatment of OS.

Accumulating research has shown that the occurrence of cancer is usually accompanied by an abnormal expression of certain miRNAs [46,47]. In OS, several miRNAs have been identified as tumor suppressors or promoters [48,49]. miR-223-3p has been shown to inhibit the metastasis and development of human OS *in vivo* and *in vitro* [50]. In addition, upregulation of miR-186-5p resulted in a significant inhibition of the malignant biological behavior of OS cells [49]. miR-23b-3p was found to be downregulated in hepatocellular carcinoma and gastric cancer but upregulated in renal cancer and OS [24–28]. Previous studies suggest that miR-23b-3p acts as either a tumor promoter or tumor suppressor in different cancers. Ran Zhu1 *et al*. (2019) reported a study demonstrating that miR-23b-3p knockdown had an obvious inhibitory effect on the proliferation of OS *in vitro* and *in vivo* [28]. Similar results were obtained *in vitro* in this study: the addition of an exogenous miR-23b-3p mimic significantly improved cell viability, proliferation, and migration of OS cells. Compared with previous studies on miR-23b-3p in OS [28,51,52], our study confirmed the promoting effect of miR-23b-3p on the viability and proliferation in OS. In addition, we showed using a wound-healing assay that miR-23b-3p promoted the migration of two OS cell lines, which is novel and different from the findings of previous studies.

VEPH1 is a protein-encoding gene located on chromosome 3q24-26 [53]. According to relevant reports, this region is amplified in a wide spectrum of human cancers, such as esophageal carcinoma, lung cancer, ovarian cancer, and cervical cancer [13,53]. It has been reported that VEPH1, as an endogenous regulatory protein, regulates multiple signaling pathways, thereby affecting tumor progression [17]. Other researchers have reported that VEPH1 is abnormally expressed in a variety of cancers. For example, Shathasivam e*t al*. [53] found that VEPH1 was upregulated in three human epithelial ovarian cancer cells, suggesting its oncogenic role in ovarian cancer. In addition, a study on OS cells showed that VEPH1 might negatively regulate the invasion and proliferation of OS cells via upregulation of cytochrome c oxidase subunit II (COX2) [54]. Consistent findings were obtained in this study, in which VEPH1 was found to be downregulated in OS tissues as well as cells and VEPH1 acted as a tumor suppressor in OS cells. We also showed that VEPH1 is the target gene of miR-23b-3p and showed an inverse relationship with miR-23b-3p. It could also reverse the promoting effect of miR-23b-3p on the progression of OS.

Although our experiments demonstrated that miR-23b-3p accelerated OS cell migration by targeting VEPH1, our study has some limitations, such as the lack of animal studies and limited clinical samples. In follow-up research, we need to confirm the role and mechanism of miR-23b-3p on OS in living animals and to collect more tumor samples to investigate the clinical significance of miR-23b-3p.

The PI3K/AKT signaling pathway is a key driver of OS tumorigenesis. It can promote OS proliferation and inhibit apoptosis and is considered the most attractive target for anti-OS drug development [31,34]. It has been demonstrated that miRNAs in OS inhibit mRNA translation and regulate PI3K/AKT signaling pathway activation at the post-transcription level [55]. In addition, previous studies have shown that VEPH1 reduces AKT activation [12]. Furthermore, miR-23b-3p interference decreased p-PI3K and p-AKT levels, whereas miR-23b-3p overexpression increased them [56]. In this study, similar to previous reports, miR-23b-3p overexpression significantly increased PI3K and AKT phosphorylation in OS cells, whereas upregulation of VEPH1 inhibited PI3K/AKT signaling. Additionally, the PI3K inhibitor LY294002 promoted OS cell apoptosis and inhibited proliferation [34]. In this study, we found that inhibition of the PI3K/AKT signaling pathway regulated by miR-23b-3p/VEPH1 axis impaired the proliferation and migration of OS cells. Therefore, we concluded that miR-23b-3p was found to promote OS by targeting VEPH1 and activating the PI3K/AKT signaling pathway.

## Conclusions

In summary, this study preliminarily demonstrated that miR-23b-3p, as a cancer promoter, interacted with VEPH1/PI3K/AKT in OS and facilitated the malignant phenotypes of OS cells. The study also showed that miR-23b-3p could inhibit VEPH1 expression in OS by binding to the VEPH1 3ʹ-UTR and activating the PI3K/AKT signaling pathway. Thus, the findings of this study indicate new potential mechanisms involved in the progression of OS.

## Supplementary Material

Supplemental Material

## Data Availability

The datasets used and/or analyzed during the current study are available from the corresponding author on reasonable request.

## Highlights:

- miR-23b-3p was upregulated in osteosarcoma and prominently promoted the viability, proliferation, and migration of osteosarcoma cells.
- VEPH1 expression was decreased in osteosarcoma, and it inhibited the viability, proliferation, and migration of osteosarcoma cells.
- VEPH1 is a downstream target gene of miR-23b-3p.
- miR-23b-3p targets VEPH1 and promotes osteosarcoma via PI3K/AKT pathway.
