# Supplementary material for: MicroRNA miR-23b-3p promotes osteosarcoma by targeting ventricular zone expressed PH domain-containing 1 (VEPH1)/phosphatidylinositol 3-kinase/protein kinase B (PI3K/AKT) pathway
Source: Bioengineered. 2021 Dec 14;12(2):12568–82. doi: 10.1080/21655979.2021.2010383 (PMC8810025; doi:10.1080/21655979.2021.2010383)
Supplement: Supplemental Material [file KBIE_A_2010383_SM9102.zip › supplementary/Supplementary Table 1_revised.docx]

Table 1. Clinical characteristics of 24 cases osteosarcoma patients.

| characteristics | N=24 | miR-23b-3p expression | | P value |
| --- | --- | --- | --- | --- |
|  |  | Low (N=12) | High (N=12) |  |
| Age(years) |  |  |  | 0.400 |
| ≥11 | 9 | 6 | 3 |  |
| <11 | 15 | 6 | 9 |  |
| Gender |  |  |  | 0.414 |
| Male | 11 | 4 | 7 |  |
| Female | 13 | 8 | 5 |  |
| Location |  |  |  | 0.241 |
| Distal femur | 11 | 7 | 4 |  |
| Proximal tibia | 6 | 2 | 4 |  |
| Proximal humerus | 4 | 3 | 1 |  |
| Proximal femur | 2 | 0 | 2 |  |
| Other | 1 | 0 | 1 |  |
| Recurrence |  |  |  | 0.037 |
| Yes | 5 | 0 | 5 |  |
| No | 19 | 12 | 7 |  |
| Metastasis |  |  |  | 0.012 |
| Yes | 13 | 3 | 10 |  |
| No | 11 | 9 | 2 |  |
| Death |  |  |  | 0.036 |
| Yes | 10 | 2 | 8 |  |
| No | 14 | 10 | 4 |  |
| WHO classification |  |  |  | 0.080 |
| Osteoblastic | 11 | 8 | 3 |  |
| Chondroblastic | 9 | 2 | 7 |  |
| Fibroblastic | 4 | 2 | 2 |  |
